# Rapid Methods for Assessing Food Safety and Quality

**DOI:** 10.3390/foods9040533

**Published:** 2020-04-23

**Authors:** Pierina Visciano, Maria Schirone

**Affiliations:** Faculty of Bioscience and Technology for Food, Agriculture and Environment, University of Teramo, 64100 Teramo, Italy; pvisciano@unite.it

**Keywords:** safety, assay, pollutants, polyciclic aromatic hydrocarbons, *Trichinella*, *Anisakis*, *Listeria monocytogenes*, nanoparticles

## Abstract

Food safety represents a central issue for the global food chain and a daily concern for all people. Contaminated food by physical, biological or chemical hazards can harm consumers, increasing demand for health services, government expenditure on public health and other social costs. The quality assurance programs are based on the continuous monitoring of raw matter, production process, storage and distribution of the end products, including the purpose for which they are intended. Such programs represent an important objective for food producers, not only for the potential risk to human health, but also for the economic losses to which they can be subjected. The development and use of rapid analytical methods able to identify the main failures in food production can benefit food companies by saving time and costs for the good and fast control of products through the entire food chain.

Nowadays, food safety has a critical societal importance for producers, regulatory control bodies and consumers. For this purpose, the food industry requires fast, sensitive, reliable, cost-effective and easy-to-use analytical techniques to assess both the safety and quality of products [1,2]. In the last few years, the traditional culture-based enumeration tests used for the detection of microorganisms have become obsolete for real-time applications, as they are time-consuming and labor-intensive, while immunological assays such as the enzyme immunoassay, enzyme-linked immunosorbent assay, enzyme-linked fluorescent assay, flow injection immunoassay and other serological methods are known for their speed, high-throughput capacity and possibility of precise quantification of the target organism [3]. 

Even for chemical analysis, the development of quick and easy methods is beginning to be preferred to classical laborious techniques [4]. One of these pre-treatment assays for sample preparation is based on the QuEChERS method, with the advantages summarized in its acronym (quick, easy, cheap, effective, rugged, and safe). The study of Nagyová and Tölgyessy [5] reported the validation of a rapid and non-laborious method for the determination of selected H_2_SO_4_ stable halogenated priority pollutants (eight organochlorines and six polybrominated diphenyl ethers) in nine different fish species. A modified QuEChERS sample preparation followed by gas chromatography-triple quadrupole tandem mass spectrometry analysis was described. In particular, the applied method showed some advantages in terms of simplicity, rapidity, high extract clean-up efficiency and good sensitivity. Moreover, the proposed assay can be classified as “an acceptable green analysis method” with low consumption of hazardous solvents. 

Puljić et al. [6] evaluated the differences in polyciclic aromatic hydrocarbons (PAHs) content in samples of traditional dry cured pork meat products made in Herzegovina, subjected to uncontrolled (traditional smokehouse) and industrial smoking processes. The results highlighted that the use of traditional smoking methods resulted in higher PAH contamination than the industrial one. At the end of production, the inner parts of all smoked samples produced using both methods retained significantly lower total PAH concentration, as well as less individual PAHs than the surface layer. The amount of the four priority PAHs in samples subjected to traditional smoking highly exceeded maximum limits (12 µg/kg) set by the Commission Regulation (EU) No 835/2011 [7] by up to 10 times. The results of this study indicate that, in order to decrease the level of PAHs and reduce the risk of PAH occurrence in smoked meat products, local producers should learn how to use the improved/novel smoking techniques and adjust the smoking parameters.

Chen [8] studied the correlation between water activity (Aw) and moisture content (MC) of floral honey at 10 and 30 °C using the Aw method. It was observed that the temperature significantly affected the Aw/MC data, but no universal linear equation for these parameters could be established. In this study, the slope was affected only by the state of the honey (liquid or crystallized), while the other factors, such as type, geographical collection sites and botanical source did not significantly affect the slope, but only the intercept of the Aw equation. The results demonstrate that such a linear equation could be used to express the relationship between Aw and MC of honeys.

Among the food biological hazards, the presence of parasites is disgusting and dangerous at the same time. The paper by Schirone et al. [9] described the accreditation procedure and the requirements for its maintenance applied by an internal laboratory attached to a slaughterhouse for the detection of *Trichinella* spp. in swine carcasses. The main advantages were represented by the possibility to analyze the muscle samples quickly, in order to obtain fast results and process carcasses after a short time from slaughtering. On the other hand, the main difficulties shown by the technician working in the internal laboratory were the structural features required by the accreditation body, i.e., the proficiency testing to be carried out each year and the training courses to be followed in order to maintain the accredited status. However, the possibility of performing the regulatory investigation of *Trichinella* spp. in swine and other species susceptible to the parasite inside the slaughterhouse is useful for customers as well as for the competent authority in guaranteeing the safety of the slaughtered carcasses. 

The study of Cammilleri et al. [10] described a rapid, reliable and easy assay for the detection of *Anisakis* spp. Such a method based on loop-mediated isothermal amplification (LAMP) was optimized, validated and applied in processed fish samples artificially contaminated with the parasite, giving sensitivity values equal to 100%. Indeed, the specificity test provided no amplification for other similar genera of parasites, i.e., *Contracaecum*, *Pseudoterranova*, or *Hysterothylacium*, as well as for uninfected samples. Moreover, the LAMP assay showed both a lower limit of detection and time of analysis in comparison with the real-time PCR method also used in this study, and its ease of use suggested that it could be a valid alternative for routine examination to help manufacturers in HACCP (Hazard Analysis Critical Control Point) application in the fishery sector. 

Among the key attributes of a microbial detection system, there are sensitivity, i.e., the ability to find the target microorganism at low contamination level, and specificity, which allows the selective detection of the target microorganism without cross-reaction with other species. However, rapidity is also a good feature of an analytical assay to obtain fast and reliable results [11]. The nucleic acid-based techniques aim to detect the specific nucleic acid sequence by amplifying the target sequence to millions of folds. They usually provide more timely and accurate results compared with traditional immunoassays and culturing methods [12]. The study of Torresi et al. [13] applied a specific real-time PCR screening assay designed for the rapid identification of *Listeria monocytogenes* strains potentially related to the outbreaks occurred in Central Italy from January 2015 to March 2016. A total of 37 clinical strains isolated from patients exhibiting listeriosis symptoms and 1374 strains correlated to the outbreak were examined by the Italian National Reference Laboratory for this microorganism with a first screening assay using a specific real-time PCR. The rapid applied method was able to quickly screen up to 96 strains in 2–4 h and to identify all strains linked to the outbreak. Then, the PCR-positive strains were subsequently typed by other tests and precisely pulsed field gel electrophoresis and next generation sequencing. The results highlight the decrease in the time and costs of analysis, as well as the importance of the rapid identification of contaminated food and food processing industries to stop the diffusion of pathogenic strains and minimize the number of cases in a foodborne outbreak. 

In recent decades, novel rapid and non-destructive methods for evaluating food quality attributes have been developed. In particular, new detection assays have been explored such as biosensors and novel nanotechnology-based immunoassay are quickly replacing traditional methods offering some advantages to food business operators. Unlike conventional methods, these techniques acquire data without contact with samples, and some examples are computer vision, spectral or hyperspectral imaging, ultrasound, near-infrared spectroscopy (NIR), Fourier transform near infrared spectroscopy (FT-NIR), and Raman spectroscopy [14]. Basile et al. [15] used a fast, simple and non-destructive technique, namely FT-NIR spectroscopy combined with chemometrics, applied to search for a correlation between the chemical composition in terms of sugar and acidic content and sensory data. Two very different table grape varieties were investigated by chemical (total soluble solid content, titratable acidity, and HPLC analysis of organic acids), FT-NIR and sensory analyses. The results provide a good basis for more elaborate studies to correlate sensory properties and chemical composition of food. 

Recently, the use of nanosensors showed the advantages of high efficiency, rapidity and sensitivity, and therefore they were applied for clinical diagnosis, pesticide residue analysis and microbiological assays. Bi et al. [16] reported a study about the detection of histamine, a spoilage monitoring compound for distinguishing the lifetime and freshness of fishery products, by using gold nanoparticles (Au-NPs) with dual approaches of colorimetric and fluorescence perspectives. The Fourier transforms infrared (FTIR) spectra provided histamine concentrations in spiked salmon muscle samples with recovery values ranging from 96 to 103%. Besides high sensitivity and selectivity, such a system was characterized by the simple pre-treatment of samples, suitable equipment requirements and low costs, so that it could be used for freshness and spoilage determination of fish, as well as for safety assurance purposes. 

To conclude, the present Special Issue consists of eight papers on various food matrices analyzed by fast, cheap and reliable techniques, sometimes as an alternative to the recognized official methods. Most of them aimed at verifying food safety as a fundamental attribute for the food processing industry, food retailers and distributors, and competent authorities due to the potential direct impact on consumer health.
